# Spatial sequestration of activated-caspase 3 in aggresomes mediates resistance of neuroblastoma cell to bortezomib treatment

**DOI:** 10.1038/s41598-024-54140-7

**Published:** 2024-02-14

**Authors:** Kévin Berthenet, Eliézer Aïmontché, Sara El Mrini, Johan Brière, Nathalie Pion, Isabelle Iacono, Stéphanie Brejon, Karine Monier, Frédéric Catez, Gabriel Ichim, Valérie Combaret, Hichem C. Mertani, Jean-Jacques Diaz, Marie Alexandra Albaret

**Affiliations:** 1 Univ Lyon, Université Claude Bernard Lyon 1, INSERM U1052, CNRS UMR5286, Centre Léon Bérard, Cancer Research Center of Lyon, 69008 Lyon, France; 2 Department of Translational Research and Innovation, Centre Léon Bérard, 69373 Lyon, France; 3 Institut Convergence PLAsCAN, 69373 Lyon Cedex 08, France; 4 DevWeCan Labex Laboratory, 69373 Lyon Cedex 08, France

**Keywords:** Cancer, Cell biology, Molecular biology

## Abstract

Neuroblastoma (NB) is the most common pediatric tumor and is currently treated by several types of therapies including chemotherapies, such as bortezomib treatment. However, resistance to bortezomib is frequently observed by mechanisms that remain to be deciphered. Bortezomib treatment leads to caspase activation and aggresome formation. Using models of patients-derived NB cell lines with different levels of sensitivity to bortezomib, we show that the activated form of caspase 3 accumulates within aggresomes of NB resistant cells leading to an impairment of bortezomib-induced apoptosis and increased cell survival. Our findings unveil a new mechanism of resistance to chemotherapy based on an altered subcellular distribution of the executioner caspase 3. This mechanism could explain the resistance developed in NB patients treated with bortezomib, emphasizing the potential of drugs targeting aggresomes.

## Introduction

Neuroblastoma (NB) is a major pediatric neoplasia of the extracranial neuroectodermal tissue, affecting approximately 1/100,000 children each year, mostly toddlers, and accounting for about 10% of childhood solid malignancies^1^. The median age at diagnosis of NB is 17 months, with 40% of patients diagnosed at infancy and 90% of patients before 10 years of age^2^. A major clinical issue resides in the fact that approximately 70% of cases are metastatic at diagnosis^3^. NB is a malignant tumor that originates from the embryonic autonomous nervous system and arises in tissues of the sympathetic nervous system, typically in the adrenal medulla or paraspinal ganglia. It can thus be present anywhere along the sympathetic chain as mass lesions in the neck, chest, abdomen, or pelvis^4^. The predominant localization of NB is the abdomen and adrenal glands^3^. NB is unique because of its clinical bipolarity: NB is associated with high morbidity and mortality rates among children, albeit it displays one of the highest levels of spontaneous and complete regression among human cancers^4^. NB is a complex disease with several well-known genetic alterations including amplification of *MYCN* and mutations in *ALK*^5^. It was reported that high *MYCN* expression confers opposite biological effects whether it is associated with gene amplification or not, the former being of poor prognosis, whereas the latter is associated with a favorable outcome^6^. In infants with a favorable biology, many tumors including metastatic NB spontaneously regress without treatment, even in the event of the metastatic diseases. Conversely, for toddlers older than 18 months of age at diagnosis, with metastatic NB and a *MYCN* amplification status, intensive multidisciplinary therapy is required including chemotherapy, surgery, radiotherapy and autologous hematopoietic stem cell transplantation^5^. Although these therapies are efficient for NB treatment in most cases, the management of high-risk patients remains particularly delicate, and the identification of new therapies is thus decisive for such patients. Among potential chemotherapies, bortezomib was proposed as a novel therapeutic agent in pediatric oncology and particularly in NB in the last decades^7,8^. Indeed, promising preclinical studies prompted NB clinical trials with bortezomib in association with conventional chemotherapy^9,10^.

Bortezomib, a dipeptidyl boronic acid, was shown to display antitumor effects through its capacity to induce cell cycle arrest and block survival factor production via inhibition of NF-κB activation. Indeed, bortezomib prevents degradation of its inhibitor IκB in cytoplasm, thus limiting translocation of NF-κB into nucleus where it can stimulate the expression of genes involved in cell proliferation and resistance to induction of apoptosis^11^. Bortezomib could also induce apoptosis by efficiently activating a class of proteases called caspases^12^. Caspase activation is a crucial event in apoptosis. These proteases are classified in two groups according to their function in the apoptotic process: the initiator caspases, caspases 8 and 9, and the executioner caspases. Caspase-3 (Casp3) is synthesized as an immature zymogen composed of a N-terminal prodomain, a large and a small subunit requiring specific proteolysis to be activated. Indeed, the prodomain is removed and the catalytic domain is cleaved to a large and a small subunit leading to the activated form of Casp3, Casp3A^13,14^. Casp3 is one of the main executioner caspases, the activation of which leads to a cascade of events resulting in DNA fragmentation, destruction of the cytoskeleton and nuclear proteins, such as the Poly-ADP-ribose polymerase 1 (PARP-1), and finally to the formation of apoptotic bodies followed by rapid cell death^15^. More precisely, activated Casp3 (Casp3A) cleaves several vital cellular proteins including PARP-1, a protein involved in DNA repair^16^. Increased membrane permeability during apoptosis allows Casp3A to enter the nucleus by simple diffusion where it cleaves PARP-1^17^ into two fragments, resulting in its inactivation and driving later phases of apoptosis including DNA fragmentation^18^. The first fragment of 24 kDa contains the N-terminal DNA-binding domain, whereas the second 89 kDa contains the C-terminal catalytic fragment. Despite the presence of Casp3A in some patients-derived NB cell lines studied^19^, they were reported to develop resistance to bortezomib^20^, the mechanisms of which remain to be deciphered.

Moreover, bortezomib was shown to bind with high affinity and reversibly inhibit the 26S proteasome, a large protease complex responsible for most protein degradation in the cytosol and the nucleus^21^. Proteasome inhibitors induce the accumulation of unfolded proteins^22^. This cellular mismanagement of aggregation-prone proteins in turn induces their accumulation within aggresomes^23^. Cellular aggresomes are highly dynamic structures built as alternative pathways to actively store misfolded proteins when the proteasome is inhibited or overwhelmed, a sort of “storage bin”. Regulation of aggresome biogenesis requires an intact microtubule network and transport of misfolded polypeptides, such as working dynein motor-proteins and histone deacetylase 6 (HDAC6). Misfolded proteins are transported along microtubules to the microtubule organizing center (MTOC). Aggresome formation is accompanied by a rearrangement around the MTOC of several cytoskeletal proteins including vimentin (Vim). Vimentin filaments form a « cage » that stabilizes the aggregated proteins over time^24^. Autophagy, mechanism of cytoplasmic component degradation^25^, subsequently contributes to the degradation of the aggregated proteins via lysosomes^26^. Aggresome formation was initially observed in neurodegenerative diseases^27^ and more recently in cancer^28–30^. The consequences of aggresome accumulation on cellular fate are still debated: aggresomes could be neurotoxic by inducing apoptosis^31^ or could be neuroprotective by ensuring cell survival^32^. The objective of the study was to determine the putative impact of aggresome formation on apoptosis execution in bortezomib-treated NB cells. We hypothesized that during the treatment of NB cells with bortezomib, the formation of aggresomes occurs, leading to the accumulation of a large proportion of Casp3A. We confirmed this hypothesis in resistant patients-derived NB cells, and observed that Casp3A aggregation limited its interaction with PARP-1 and compromised apoptosis execution. Hence, spatial sequestration of Casp3A within aggresomes constitutes a novel cellular mechanism to counteract bortezomib-induced apoptosis, favoring resistance to chemotherapy.

## Results

### Despite similar Casp3/7 activation rates, the growth and proliferation of NB cells are differently impacted by bortezomib treatment

**Figure 1:**
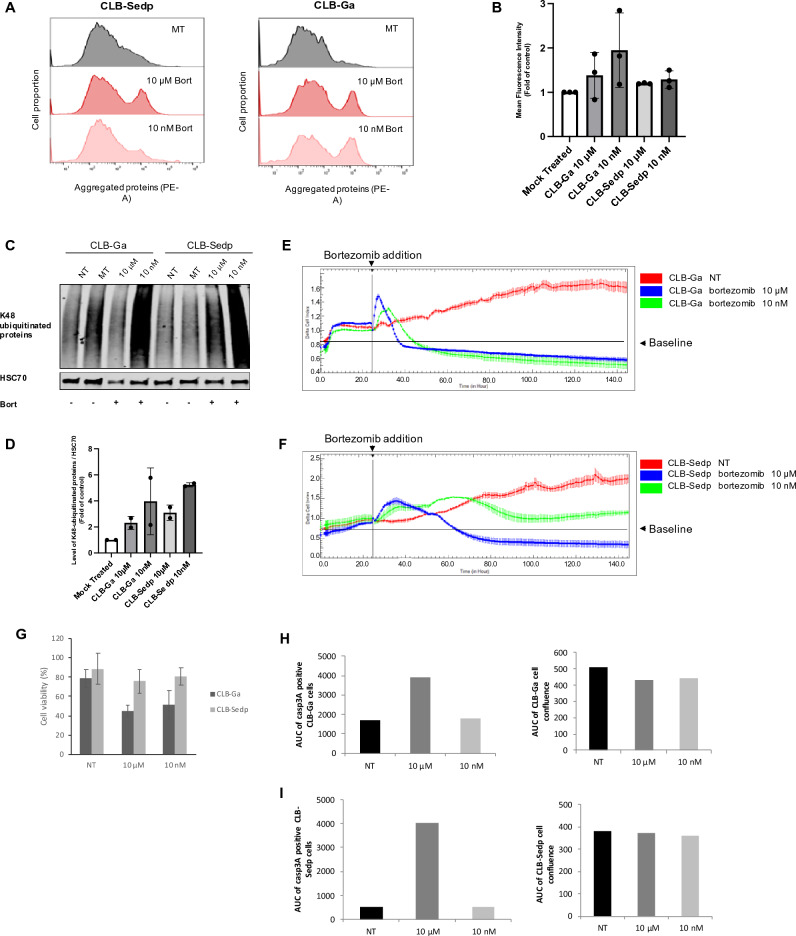
Distinct response of NB cell lines to bortezomib treatment. (**A**) Accumulation of aggregated proteins in CLB-Ga and CLB-Sedp cells treated or not with 10 nM or 10 μM bortezomib during 24 h was analyzed by FACS using the proteostat detection kit (ref ENZ-51035-0025). (**B**) Quantification of mean fluorescence intensity and normalization by the mock treated condition (n = 2 ± SEM). (**C**) 20 μg of whole protein lysates from CLB-Ga and CLB-Sedp cells treated or not with 10 nM or 10 μM bortezomib for 24 h were separated by SDS-PAGE electrophoresis and transferred onto nitrocellulose membranes. Detection of K48 ubiquitinated proteins by anti-K48-linkage Specific Polyubiquitin mAb. HSC70 was used as a loading control. Original blots are included in supplementary Fig. 1. (**D**) Quantification of K48 ubiquitinated proteins was conducted using Image Studio Lite Version 5.2 software. Histogram representing the K48 ubiquitinated proteins signal intensity normalized against HSC70 (n = 2 ± SEM). (**E**,**F**) The growth and proliferation of CLB-Ga and CLB-Sedp cells treated or not with 10 nM or 10 μM bortezomib was measured for 140 h by xCELLigence (n = 1). (**G**) Viability of CLB-Ga and CLB-Sedp cells was evaluated by trypan blue exclusion. Cells were treated or not with 10 nM or 10 μM bortezomib for 24 h (n = 2 ± SEM). (**H**,**I**) Kinetics of caspase-dependent apoptosis and cellular confluence using the IncuCyte ZOOM^®^ technology. CLB-Ga (D) and CLB-Sedp (E) cells were treated or not with 10 nM or 10 μM bortezomib for 24 h. Casp3/7-positive cells per surface (mm^2^) (left panel) were counted and cell confluence (%) (right panel) was measured. Histograms representing the integration of the area under the mean curve (triplicate of three wells per condition) over 24 h. AUC: Area Under Curve (n = 1). For all panels NT: Not treated; MT: Mock treated.

We used two patients-derived NB cell lines, namely CLB-Ga and CLB-Sedp, derived from surgical resection (Centre Léon Bérard, CLB comprehensive cancer center). We first checked that, as expected, bortezomib treatment has an impact on the proteostasis of the cells. As shown in Fig. 1A,B, compared to the mock treated condition, bortezomib induced the accumulation of aggregated proteins and increased the accumulation of K48 ubiquitinated proteins (Fig. 1C,D). The growth and proliferation of these cells treated with 10 µM or 10 nM bortezomib was analyzed continuously over 140 h using an impedimetric system (xCELLigence system). As shown in Fig. 1E, the delta cell index values of CLB-Ga cells treated with 10 µM or 10 nM bortezomib exceeded, at 16 h and 24 h post-treatment respectively, the baseline, extrapolated from values of non-treated cells at t = 0. These values indicated a clear arrest in cell growth and proliferation at both concentrations. In comparison, CLB-Sedp cells displayed a 40 h delay in the inhibition of cell growth and proliferation when treated with 10 µM bortezomib, and showed an intermediate phenotype at 10 nM of treatment, with a continuous growth albeit at a lower rate than untreated cells (Fig. 1F). These results revealed that CLB-Ga and CLB-Sedp cells do not display the same level of sensitivity to bortezomib. In addition, we observed a decrease in cell viability by trypan blue exclusion exclusively for CLB-Ga cells after 24 h of bortezomib treatment (Fig. 1G). As it was previously reported that bortezomib treatment triggers apoptosis^19^, we monitored Casp3/7 activation following bortezomib treatment during 24 h by IncuCyte-based live cell imaging. This technology allows the detection of cells presenting an enzymatically active form of Casp3/7 (Casp3/7A). As expected, the number of Casp3/7A-positive cells increased slightly when bortezomib was added at 10 nM and very strongly at 10 μM. Treatment with bortezomib at a high concentration induced a similar rate of Casp3/7 activation in CLB-Ga and CLB-Sedp cell lines (Fig. 1H,I left panel). In parallel, we observed a higher inhibition of cell confluence for treated CLB-Ga cells than for treated CLB-Sedp cells (Fig. 1H,I right panel), indicating a discrepancy in the rate of cell confluence between the two cell lines at this concentration.

Taken together, these results highlight that despite a similar level of induction of apoptosis in CLB-Ga and CLB-Sedp cells, cell fate determination of the two cell lines was radically different, with CLB-Ga being highly sensitive while CLB-Sedp cell displaying a resistance to bortezomib.

### Casp3 and aggresomes are higher in resistant CLB-Sedp NB cells

**Figure 2:**
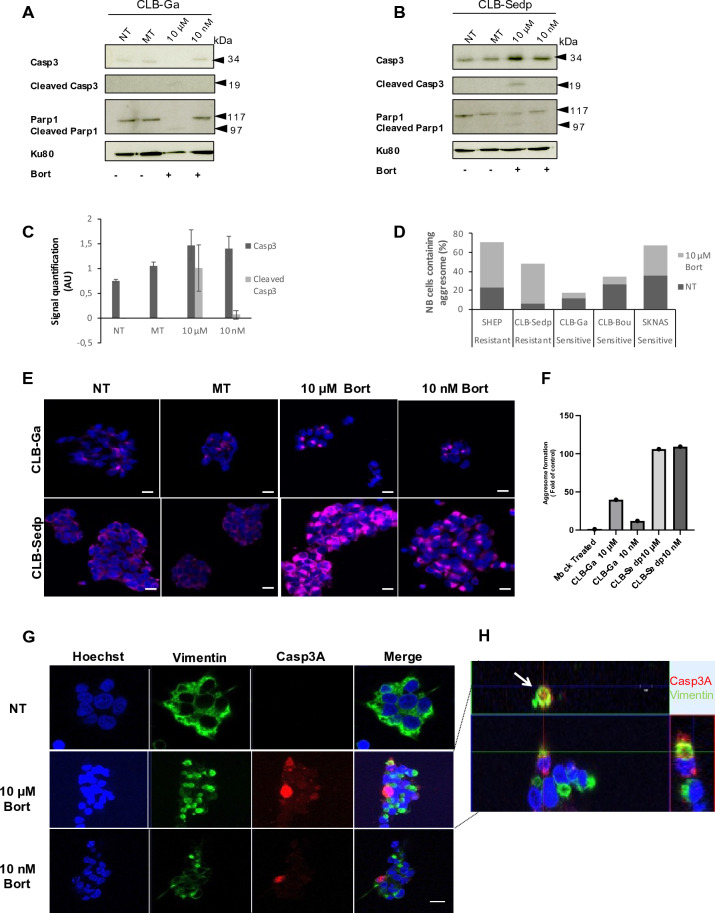
Casp3A accumulates in aggresomes of resistant CLB-Sedp cells. (A,B) 15 μg of whole protein lysates from CLB-Ga (**A**) and CLB-Sedp (**B**) cells treated or not with 10 nM or 10 μM bortezomib for 24 h were separated by SDS-PAGE electrophoresis and transferred onto nitrocellulose membranes. Detection of Casp3 and PARP-1 by anti-Casp3 mAb and anti-Parp1 mAb, respectively. Ku80 was used as a loading control. Original blots are included in supplementary Fig. 2. (**C**) For CLB-Sedp cells: quantification of Casp3 was conducted using Image J software. Histogram representing the Casp3 signal intensity normalized against Ku80 (n = 2 ± SEM). (**D**) SHEP, CLB-Sedp, CLB-Ga, CLB-Boult and SKNAS cells treated or not with 10 μM bortezomib for 24 h were analyzed by immunofluorescence (IF). Cells were fixed, permeabilized and stained for vimentin. The number of vimentin cages corresponding to aggresomes was counted by microscopic observation of 10 random fields. Histograms represent the percentage of cells containing aggresomes. (**E**) IF analysis by confocal microscopy of vimentin subcellular localization in CLB-Ga and CLB-Sedp cells treated or not with 10 μM or 10 nM bortezomib during 24 h. Fluorescence micrographs of vimentin cages (anti-vim antibody, red signal) on fixed and permeabilized CLB-Ga and CLB-Sedp cells. Hoechst dye was used to stain the nuclei (blue) (scale bar = 10 μm). (**F**) Quantification of vimentin cages by 10 random fields in CLB-Ga and CLB-Sedp cells. Histograms represent the normalized value of aggresome formation. (**G**) IF analysis by confocal microscopy of Casp3A and vimentin subcellular localization in CLB-Sedp cells treated or not with 10 nM or 10 μM bortezomib for 24 h. Fluorescence micrographs of Casp3A (anti-Casp3A antibody, red signal) and Vimentin (Anti-vimentin mAb, green signal) on fixed and permeabilized cells. Hoechst dye was used to stain the nuclei (blue) (scale bar = 20 μm). (**H**) Superposition of green and red signals is presented on enlarged views of the merged images (white arrow). For all panels NT: Not treated; MT: Mock treated.

Caspases are present in cells as inactive zymogens also known as procaspases. Procaspase 3 is present in the form of constitutive dimers. Cleavage within the inter-subunit linker region and release of large and small subunits induces activation after their dimerization^33^. Western blot (WB) analysis confirmed Casp3 activation by highlighting the Casp3 precursor and its cleaved form (19 kDa C terminal fragment), as well as the cleavage of its substrate PARP-1 after 24 h of bortezomib treatment (Fig. 2A). As expected, we observed in CLB-Ga cells treated with 10 μM bortezomib a complete loss of procaspase 3 associated with an increase in the cleaved Casp3 fragment. In turn, PARP-1 was totally cleaved and an increased accumulation of the 89 kDa cleaved fragment was observed (Fig. 2A). Conversely, the accumulation of cleaved Casp3 in CLB-Sedp cells treated with 10 μM bortezomib did not lead to a loss of procaspase 3 but to an increase in its accumulation (Fig. 2B). This was also observed for PARP-1 and cleaved PARP-1 (Fig. 2B). Quantification of procaspase 3/cleaved Casp3 confirmed a higher rate of both forms in 10 μM bortezomib CLB-Sedp-treated cells compared to NT or MT cells (Fig. 2C). These results suggest that the mechanisms of Casp3 activation are disrupted leading to failed PARP-1 cleavage. As it has been described that bortezomib treatment enhances protein aggregation by inhibiting proteasomes, we sought to determine whether aggresome formation occurred in bortezomib-treated NB cells. By using a panel of sensitive and resistant NB cell lines including CLB-Ga and CLB-Sedp, we scored cells presenting aggresomes (characterized by the formation of vimentin (Vim) cages) following bortezomib treatment. As shown in Fig. 2D, there was a clear increase in the number of aggresomes in resistant NB cells treated with 10 μM bortezomib. Inversely, aggresome formation was reduced by bortezomib treatment in sensitive NB cell lines (Fig. 2D).

Figure 2E shows the vimentin cage formation visualized by IF analysis in the sensitive CLB-Ga cells and in resistant CLB-Sedp cells treated by bortezomib. A quantification confirmed a higher number of aggresomes in CLB-Sedp cells compared to CLB-Ga cells (Fig. 2F).

We then determined whether accumulation of Casp3A could arise in aggresomes thus limiting Casp3 activation. By immunofluorescence and confocal microscopy, we observed the accumulation of Casp3A in treated CLB-Sedp cells (Fig. 2G) and the co-localization of Vim and Casp3A. An orthogonal confocal microscopy view highlighted that accumulation of Casp3A was observed inside aggresomes in 10 μM bortezomib-treated cells (Fig. 2H, white arrow). Collectively, these results show that Casp3 activation, as well as the subcellular localization of Casp3A, are altered in resistant CLB-Sedp cells.

### Casp3A is sequestered in aggresomes in resistant CLB-Sedp cells

**Figure 3:**
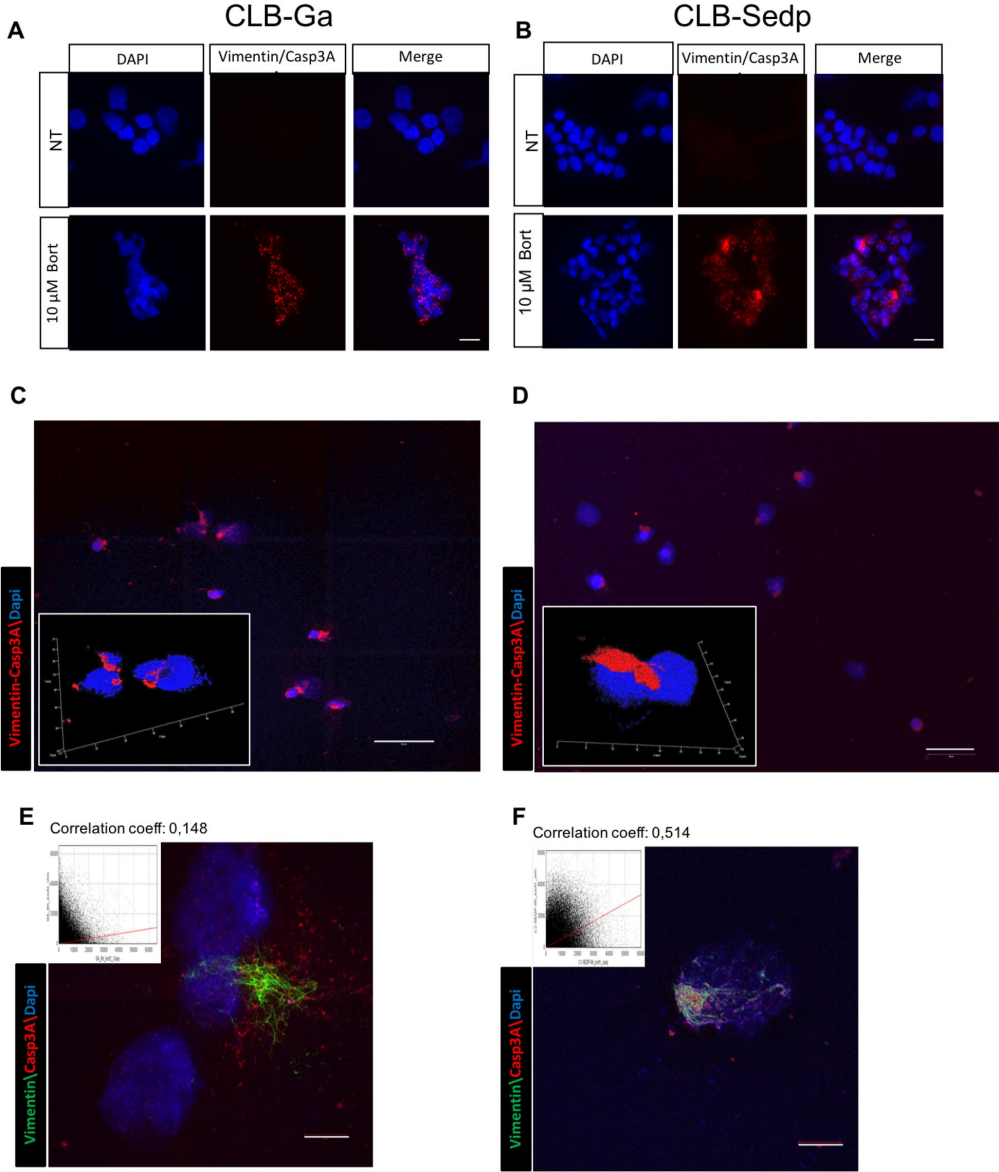
Casp3A is closely associated with vimentin cages in resistant CLB-Sedp cells. (A–F) CLB-Ga and CLB-Sedp cells treated or not with 10 μM bortezomib for 8 h. (A,B) PLA analysis of Casp3A/vimentin interactions by epifluorescence microscopy in CLB-Ga (**A**) and CLB-Sedp (**B**) cells. In red, Casp3A/vimentin interactions. In blue, DAPI-stained nuclei. NT: Not treated. (scale bar = 20 μm) (n = 1) (C,D) PLA analysis of Casp3A/vimentin interactions by confocal microscopy in CLB-Ga (**C**) and CLB-Sedp cells (**D**). Fluorescence micrographs of interactions between Casp3A/vimentin (red signal). In blue, DAPI-stained nuclei (scale bar = 50 μm). 3D reconstruction of Casp3A/vimentin interactions after PLA analysis (red signal) is presented on enlarged views for CLB-Ga (C) and CLB-Sedp cells (D). (E–F) Analysis of 3D-Structured Illumination Microscopy of individual Casp3A and vimentin subcellular localizations in CLB-Ga (**E**) and CLB-Sedp cells (**F**). Super-resolution images of Casp3A (anti-Casp3A antibody, red signal) and Vimentin (Anti-vimentin mAb, green signal) on fixed and permeabilized cells. Dapi was used to stain the nuclei (blue). Co-localization of Casp3A (red signal) and vimentin (green signal) is represented by Pearson’s correlation coefficient (scale bar = 10 μm) (n = 1).

In order to confirm the physical interaction between Vim and Casp3A and subcellular localization of Casp3A in vimentin cages, we used proximity ligation assay (PLA) with a Casp3A-specific antibody. This technique allows the visualization (as dots) and quantification of protein–protein interactions within a distance of 40 nm^34^. We thus applied this technique to CLB-Ga and CLB-Sedp cells treated with 10 μM bortezomib for 8 h. Whereas, the interaction between PLA Casp3A-Vim occurred in a diffuse manner throughout the whole cytosol of CLB-Ga cells (Fig. 3A), Casp3A-Vim seemed to aggregate in a perinuclear area in CLB-Sedp cells, indicating that these interactions may take place in vimentin cages (Fig. 3B).

In addition, confocal microscopy analysis coupled to 3D reconstruction (enlarged view in Fig. 3C,D) demonstrated that Vim-Casp3A PLA signals were spread all around the nucleus of CLB-Ga cells (Fig. 3C), while in CLB-Sedp cells, Vim-Casp3A interactions formed complexes localized close to the nucleus in a perinuclear and asymmetric position similar to vimentin cages (Fig. 3D). We confirmed the co-localization of the two proteins using 3D-Structural Illumination Microscopy (3D-SIM) fluorescence imaging, providing images at the nanometric scale (Fig. 3E,F). In CLB-Sedp cells, Vim and Casp3A co-localized in a perinuclear asymmetric area, as evidenced by the high value (0.514) of the Pearson’s correlation coefficient (Fig. 3F). In CLB-Ga cells, we observed a different distribution between microfilaments of vimentin and Casp3A both having a distinct diffuse cytosolic distribution as confirmed by the low value (0.148) of the Pearson’s correlation coefficient (Fig. 3E). Taken together, these results suggest that Casp3A was trapped in aggresomes of CLB-Sedp cells, which may explain the resistant phenotype of these cells to bortezomib treatment.

### Apoptosis is disrupted in resistant CLB-Sedp cells owing to Casp3A sequestration in aggresomes

**Figure 4:**
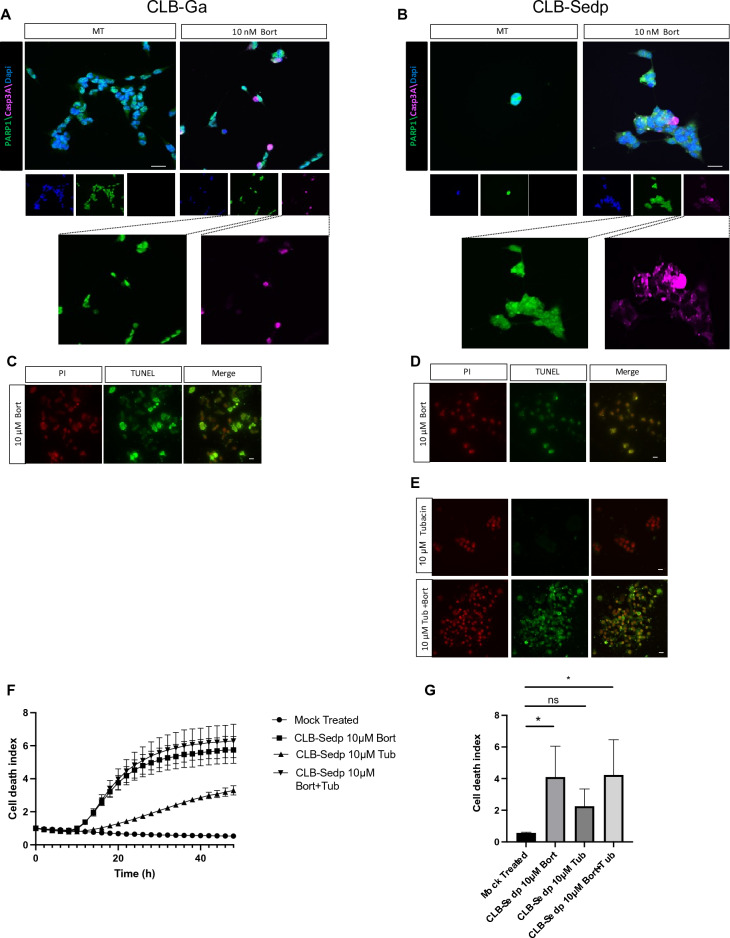
Late stage apoptosis is restored in resistant CLB-Sedp cells after treatment with the aggresome inhibitor tubacin. IF analysis of Casp3A and PARP-1 subcellular localization in CLB-Ga (**A**) and CLB-Sedp cells (**B**) treated or not with 10 nM bortezomib for 24 h. Fluorescence micrographs of Casp3A (anti-Casp3A antibody, red signal) and PARP-1(Anti-Parp1 mAb, green signal) on fixed and permeabilized cells. Hoechst dye was used to stain the nuclei (blue)MT: Mock treated. (scale = 20 μm) (n = 1). (C,D) TUNEL assay of CLB-Ga (**C**) and CLB-Sedp cells (**D**) treated or not with 10 μM bortezomib for 8 h (scale bar = 20 μm) (n = 1). (**E**) TUNEL assay of CLB-Sedp cells treated with 10 μM bortezomib associated or not with 10 μM tubacin for 8 h (scale bar = 20 μm) (n = 1). (**F**) Kinetics of cell death using the IncuCyte ZOOM^®^ technology. CLB-Sedp cells were treated or not with 10 μM bortezomib and/or Tubacin during 48 h. (**G**) Measurement of cell death induction by SYTOX™ Green staining was normalized by cell confluence (%) and by the first time point using the Incucyte 2022B Rev2 software. Histograms representing the integration of the area under the mean curve (triplicate of three wells per condition) over 48 h. AUC: Area Under Curve (n = 3) Unpaired T-test *p-value < 0.05.

Having shown failed PARP-1 cleavage in bortezomib-treated CLB-Sedp cells, we wondered whether Casp3A trapping in aggresomes may impact late stages of apoptosis. We first determined the subcellular localization of Casp3A and PARP-1 by IF. As expected, PARP-1 was located in the nuclei of treated CLB-Ga cells (Fig. 4A) alongside Casp3A (enlarged view in Fig. 4A). In contrast, in treated CLB-Sedp cells, PARP-1 was present in the nuclei (Fig. 4B), but Casp3A was excluded (enlarged view Fig. 4B).

These observations suggest that in treated CLB-Sedp cells PARP-1 was not accessible to Casp3A and reinforce previous results showing a limited PARP-1 cleavage. In order to characterize the late stages of apoptosis, we then characterized DNA fragmentation following 8 h of bortezomib treatment by TUNEL assay. As expected, in sensitive CLB-Ga cells harboring nuclear PARP-1 and Casp3A co-localization, a large number of DNA fragmented cells were observed (green cells in Fig. 4C) compared to resistant CLB-Sedp cells (Fig. 4D), confirming impairment of late stage apoptosis in these cells presenting exclusive PARP-1 and Casp3A subcellular localization (Fig. 4D). In order to restore late stage apoptosis, we then attempted to limit the accumulation of Casp3A in aggresomes. As it was previously reported that histone deacetylase HDAC6 regulates aggresome formation^35^, we used tubacin as an inhibitor of HDAC6. The addition of tubacin alone did not induce DNA fragmentation, but a large number of DNA fragmented cells were observed when tubacin was associated with bortezomib (Fig. 4E). Measure of cell death index during 48 h has confirmed that sensitization of CLB-Sedp cell line to bortezomib treatment was maintained overtime after addition of tubacin (Fig. 4F,G). Overall, these results suggest that the hijacking of Casp3A in aggresomes formed in resistant CLB-Sedp cells limits Casp3A diffusion to the nucleus and delays late stage apoptosis process.

## Discussion

The efficacy of chemotherapeutic treatments against NB is currently limited owing to the development of resistance, though the exact mechanisms remain to be elucidated. By using patients-derived NB cell lines sensitive (CLB-Ga) or resistant (CLB-Sedp) to bortezomib we deciphered a novel resistance mechanism linking Casp3A, vimentin and PARP-1. Despite the maintained proliferation and viability of resistant CLB-Sedp cells, treatment with 10 μM bortezomib induced an equivalent rate of Casp3A activity both in CLB-Sedp and CLB-Ga cells, suggesting that resistance to cell death was not due to a default of Casp3A enzymatic activity. Our previous studies on the two cell lines failed to unveil the involvement of pro-apoptotic factors such as p53, NOXA, Bad, PUMA, Bax or Bak, and of anti-apoptotic factors such as Bcl2, Bcl-xl, Mcl-1, XIAP and survivin in their distinct response to bortezomib^19^. Moreover, the mechanism underlying the impairment in the proper transmission of activated Casp3 signal remained elusive.

Here, we demonstrated by WB analysis that cleavage of procaspase 3 and PARP-1was not complete in CLB-Sedp compared to CLB-Ga cells. These results highlight an alteration of Casp3 activation and in turn of PARP-1 cleavage in the former cells. Alterations or disturbances in caspase cleavage like Casp3 can lead to a decrease in apoptosis and thus to cell survival^17^. A defect in the cleavage process could be due to either a mutation of Casp3-encoding genes, leading to a defective protein, and/or to a disruption of enzyme–substrate interaction necessary for proper enzyme cleavage. The presence of mutations remains to be determined in our NB model. However, we highlighted a greater tendency to aggresome formation in bortezomib treated CLB-Sedp cells, which could explain an alteration of enzyme–substrate interactions. Indeed, the number of aggresomes was eight times higher in CLB-Sedp (41.88%) versus CLB-Ga (4.87%) cell line. By PLA, we demonstrated that molecular interactions between Casp3A and vimentin in CLB-Sedp cells were much higher than in CLB-Ga cells. Analysis by confocal microscopy and 3D reconstruction suggested a Casp3A-containing aggresome in CLB-Sedp cells, which was then confirmed by IF and super-resolution fluorescence imaging of vimentin and Casp3A. Furthermore, IF of Casp3A and PARP-1 confirmed a subcellular compartmentalization of the enzyme and its substrate by highlighting a nuclear localization of PARP-1 and the exclusion of Casp3A. Hence, we demonstrated a spatial sequestration of Casp3A in aggresomes that could alter Casp3 activation and consecutively cleavage of its substrate PARP-1. As a consequence of the lack of PARP-1 inhibition, DNA fragmentation was observed fewer in CLB-Sedp cells compare to CLB-Ga cells. DNA fragmentation was restored in CLB-Sedp cells when tubacin, a specific inhibitor of aggresome formation was combined to the bortezomib treatment. Altogether, these results demonstrate that in CLB-Sedp cells a default of apoptosis execution is likely due to a limitation in the interaction between Casp3A and PARP-1 by Casp3A sequestration in aggresomes. Because PARP-1 is no longer inactivated by Casp3A, DNA repair-induced survival occurs, disrupting late stages of apoptosis.

Casp3 is a stable protein with a half-life between 8 and 11 h compare to Casp1 which is degraded in 9 min^36^. After activation, Casp3A should cleave a very large set of substrates and it is not rapidly inactivated by degradation that can take several hours to several days^37^. In consequences, the probability that Casp3A accumulated in aggresome after bortezomib treatment seems to be weak. However, we showed Casp3A sequestered in aggresome from 8 h of treatment. Therefore, its sequestration within the aggresome represent an additional layer of Casp3A regulation being equivalent to a degradation. However, the fine molecular mechanism by which Casp3A is addressed to aggresome remains to be determined. Casp3A could be then fully inactivated when the aggresomes were engulfed by lysosomes via autophagy. We thus highlighted a mechanism of apoptosis regulation relying on the sequestration of the pro-apoptotic factor Casp3A in aggresomes. This is also in line with the results of Wong’s group that found a regulation of autophagic aggresome clearance by the sequestration of the autophagy inhibitor mTOR in aggresomes. Indeed, the sequestration of mTOR in aggresomes may impact the kinetics of autophagy, which in turn influence the clearance of aggresomes^38^. Consequently, targeting Casp3A-containing aggresomes seems to be a promising therapeutic solution in order to restore apoptotic sensitivity in bortezomib-resistant NB cells. A recent study revealed that combining bortezomib with the HDAC inhibitor vorinostat (SAHA) that targets aggresome formation synergistically induced dramatic cell death in MYC-driven NB patients. This FDA-approved drug with in vivo validation thus provides a rationale for clinical evaluation of bortezomib, alone or in combination with vorinostat^39^. This recent study supports our hypothesis that targeting aggresomes containing Casp3A in combination with the administration of proteasome inhibitors is a promising therapeutic solution for NB treatment.

In other pathologies like neuropathology, toxicity or protective functions of aggresomes are still under debate. Several studies reported conclusions that seem contradictory. It has thus been shown that there is a positive correlation between the formation of aggresomes containing infectious protein prion and Casp3A in a murine neuronal cell line infected with prion proteins and treated with an inhibitor of proteasome, lactacystine. The authors highlighted a pro-apoptotic role for aggresomes^40^. Another study conducted in human neuronal cell line co-transfected for synphilin 1 and α synuclein, both involved in Parkinson (PD) disease, demonstrated that aggresome formation occurred more frequently in non-apoptotic neurons lacking DNA fragmentation. Conversely, the authors concluded to an anti-apoptotic function of aggresomes^41^. In light of our own results, these studies, far from being contradictory, complement each other and reinforce our results. Thus, the presence of the aggresome does not prevent the activation of caspases and in particular Casp3, but limits DNA fragmentation and the progress of apoptosis until the late stages through Casp3A sequestration. In this study we determined that in this mechanism of resistance to chemotherapy-induced apoptosis, the early steps of the apoptotic process are not inhibited but the late stages are limited.

In conclusion, we unveiled a new molecular mechanism of bortezomib resistance by NB cells that counteract chemotherapy-induced apoptosis. This mechanism is based on the spatial regulation of the key effector of apoptotic process Casp3A that is trapped in aggresomes impairing apoptosis and maintaining survival of bortezomib-treated NB cells. This molecular process constitutes a regulation of the proteostasis of Casp3A being equivalent to a degradation by proteasome. In light of our results, we propose that aggresomes containing Casp3A could be a promising new target for NB patient management.

## Materials and methods

### Cell lines and culture

The panel of neuroblastoma (NB) cell lines CLB-Ga, CLB-Sedp, CLB-Bou, SHEP, and SKNAS derived or not from patients of the Centre Léon Bérard (CLB) hospital and exhibiting different sensitivity to bortezomib treatment were obtained as previously mentioned^19^. All cell lines were grown in RPMI 1640 (Gibco-Invitrogen, Cergy-Pontoise, France) supplemented with 10% heat-inactivated fetal bovine serum (Cambrex-Biowhittaker, Emerainville, France) and 200 IU/ml penicillin, 200 μg/ml streptomycin and 2 mM L-glutamine (all reagents from Gibco-Invitrogen) at + 37 °C, under 5% CO_2_.

### Pharmacological treatments

Stock solution of bortezomib (PS-341, Velcade^®^) was obtained as previously mentioned^19^. 10 μM and 10 nM solutions were made from a stock solution of bortezomib (PS-341, Velcade^®^) in 2.6 mM buffer diluted in 0.9% NaCl. Tubacin (Sigma-Aldrich, Missouri, USA) was used at a 10 µM concentration.

### Experimental strategy

For all experiments, 24 h after seeding, bortezomib and/or tubacin were added to the medium (10 μM and 10 nM bortezomib) for cell treatment, or medium was replaced with fresh medium (Mock treated, MT). Cells were grown in these conditions for 8 h, 24 h or 48 h for cell viability analysis, Western blot analysis, immunofluorescence coupled or not to PLA or to TUNEL assay (A23210 APO-BrdUTM TUNEL Assay Kit /Invitrogen), real time analysis of caspase 3–7 activity (IncuCyte ZOOM). Finally, cells were treated for 140 h for real time analysis of cell growth and proliferation (xCELLigence).

### Cell viability analysis (cell counter CEDEX-XS–Roche)

The proportion of viable cells, which remained unstained upon addition of trypan blue in the medium, was calculated by comparing the number of non-viable (stained) cells to the total number of cells (trypan blue exclusion method).

### Real-time analysis of cell growth and proliferation by xCELLigence (ACEA Bioscience)

The real-time measurement instrument xCELLigence (ACEA Biosciences™) is based on the impedance value which is proportional to the fixation of cells on micro-electrodes. The system used to evaluate the proliferative capacity of cells is the RTCA DP system. This system uses CIM plates containing 16 wells. Wells are formed by chambers containing 10 micro-electrodes and placed at 37 °C and 5% CO_2_. When evaluating the cell growth and proliferative capacity of NB cells, CLB-Ga (15,000 cells) and CLB-Sedp (36,000 cells) cells were placed in the chambers containing complete culture medium supplemented with 10% FBS. The impedance generated by cell contact was measured every 15 min and recorded by a computer linked to the RTCA DP system. The normalized cell index value corresponding to the variation in electrical impedance was calculated by integrating the slopes of the mean curves of triplicate experiments ± SEM.

### Real-time analysis of cell death, cell confluence and caspase-3/7 activity by Incucyte Zoom system (Essen Bioscience)

Cell death, cell confluence and caspase-3/7-dependent apoptosis were measured in real-time with the IncuCyte Zoom Live-content imaging system^®^ (Essen Bioscience). Cells were placed at 37 °C and 5% CO_2_ with SYTOX™ Green (Life technologies) or caspase-3/7 apoptosis reagent (Essen Biosciences) at a final concentration of 30 nM and a 1:1,000 dilution respectively, in cell culture medium of both experimental and control wells. Cell confluence was monitored in parallel to cell death or caspase-3/7 apoptosis. SYTOX™ Green allows to identify dead cells through the emission of green fluorescence upon its binding to DNA. Concerning caspase-3/7-dependent apoptosis, it is detected by an inert non-fluorescent substrate with a caspase-3/7 recognition site and the peptide NucView 488 (Essen Biosciences). Once inside cells, activated caspase-3/7 cleaves the bond between the inert peptide and NucView 488. Free NucView 488 has a high affinity for nuclear DNA and is fluorescent in the green spectrum. Thus, caspase-3/7 activation is correlated with an increase in green fluorescent apoptotic cells. CLB-Ga (11,000 cells) and CLB-Sedp (15,000 cells) cells were seeded onto 96-well Corning plates and were imaged at × 10 magnification. Fluorescent signal (cell death and caspase-3/7 activity) and phase contrast (cell confluence) images were acquired every 2 h for 24 h to 48 h. Each condition was performed in triplicate. Integration of the area under the mean curves (triplicate) was used to quantify the percentage of cell confluence and the number of caspase-3/7-positive cells per surface (mm^2^) over 24 h. Concerning the cell death index, it was measured by normalizing SYTOX™ Green percentage area with cell confluence (% area) and the first time point using the Incucyte 2022B Rev2 software.

### Western blot analysis

In Western blot (WB) experiments, 5–15 μg of proteins from CLB-Ga and CLB-Sedp cell lysates were separated by SDS-PAGE and transferred onto a nitrocellulose membranes. The membrane was saturated with a solution of TBS-T (20 mM Tris–HCl, pH 7.0, 130 mM NaCl, 0.1% Tween 20) containing 5% milk, then incubated with various antibodies diluted in TBS-T solution containing 2.5% milk. The anti-casp3 Alexis (1:250) (ALX-804-305-C100, Enzo Life Sciences), anti-Parp1 (1:100) (AM30 Calbiochem), anti-K48-Linkage specific polyubiquitin (1:1000) (8081S, Cell Signaling), anti-HSC70 (1:1000) (sc-7298, Santa Cruz Biotechnology) and anti-Ku80 (1:2000) (ab3715 Abcam) murine monoclonal antibodies were incubated for 1 h at room temperature (RT) with the membrane. The primary antibodies were detected with anti-mouse or anti-rabbit secondary antibodies coupled to HRP.

### Immunofluorescence analysis

Glass coverslips were pre-coated with a solution of poly-L lysin (P9155 Sigma Aldrich) in culture medium at a dilution of 1:20 for 30 min at RT. To preserve membrane-associated components, non-permeabilized cells were fixed in 4% paraformaldehyde (PFA) at RT for 5 min. In contrast, permeabilized cells were fixed in 4% PFA at RT for 30 min. Membrane permeabilization was conducted by adding Triton X-100 (Sigma-Aldrich, Darmstadt, Germany) for 5 min. For protein expression analysis at the plasma membrane, a solution of phosphate-buffered saline (PBS) containing primary antibodies was incubated for 2 h with glass coverslips. For intracellular protein expression analysis, primary antibodies were added to a PBS solution containing 0.1 M bovine serum albumin (BSA), 0.3 M NaCl, 0.5% Tween-20, and 1% FBS, and then incubated with glass coverslips for 1 h at RT. Primary antibodies used were as follows: mouse monoclonal anti-vimentin clone V9 (1/100^e^) (M0725 DakoCytomation), rabbit polyclonal anti-Casp3A (1/400) (9661S Cell Signaling), mouse monoclonal anti-Parp1 (1/100^e^) (AM30 Calbiochem). Primary antibodies were detected using Alexa-488 or Alexa-555 secondary antibodies coupled to fluorophores for 1 h at RT. Cells then were incubated in a PBS solution containing Hoechst dye (Sigma-Aldrich, Darmstadt, Germany) diluted 1:10,000 in order to counterstain cell nuclei. Slides were observed by epifluorescence microscope (Nikon Nie), by confocal microscope (Confocal Zeiss 880) coupled to images treatment with Zen^®^ software. Acquisition of 3D-Structured illumination Microscopy (3D-SIM) was performed with an ELYRA PS1 (Zeiss) using a 63x (NA 1.4) objective lens equipped with an SCMOS camera and piloted with Zen Black software. Z-stacks of 30 sections were acquired using 5 translations and 5 rotations that were processed to calculated the 3D-SIM stack. Alignment was performed in affine mode with tetraSpeck beads acquired in the same conditions that the biological stack. Colocalization was performed on 3D-stacks with the JACOP Plugin^42^ from ImageJ to calculate the value of the Pearson’s coefficient. Maximum projections of 10 selected sections around the focal plane were accomplished for visualization (Fig. 3E,F).

### Proximity ligation assay by DuoLink technology (Sigma Aldrich)

Protein–protein interactions between Casp3A and vimentin in CLB-Ga and CLB-Sedp cell lines were analyzed by Proximity Ligation Assay, to visualize molecular interactions occurring within 40 nm. Detection was performed using anti-vimentin (M0725 DaKoCytomation) coupled to a PLA anti-mouse probe, and rabbit polyclonal antibody anti-activated Casp3 (9661S Cell Signaling) coupled to a PLA anti-rabbit probe. After staining with DAPI, coverslips containing fixed cells were mounted with Fluoromount aqueous mounting medium, and observed by epifluorescence microscopy (Nikon Nie) or by confocal microscopy (Confocal Zeiss 880) coupled to image treatment using the Zen^®^ software.

### Flow cytometry analysis

Protein aggregates in CLB-Ga and CLB-Sedp cell lines treated for 24 h with 10 µM or 10 nM bortezomib were analyzed using the proteostat detection kit (ENZ-51035-K100) according to the manufacturer’s intructions. Briefly, after treatment, cells were harvested and washed in PBS. Cells were then fixed in 4% PFA for 30 min at room temperature. Cells were then washed in PBS and permeabilized using 0.5% Triton in PBS for 30 min on ice. Cells were washed in PBS and incubated for 30 min with the Aggresome Red Detection Reagent (1:10,000). Cell fluorescence was analyzed using the LSR Fortessa HTS flow cytometer (BD Biosciences).

### Supplementary Information

Supplementary Figures.

## Data Availability

The datasets used and/or analysed during the current study available from the corresponding author on reasonable request.
