# Supplementary material for: Spatial sequestration of activated-caspase 3 in aggresomes mediates resistance of neuroblastoma cell to bortezomib treatment
Source: Sci Rep. 2024 Feb 14;14:3768. doi: 10.1038/s41598-024-54140-7 (PMC10866921; doi:10.1038/s41598-024-54140-7)
Supplement: Supplementary file 1 — Supplementary Figures. [file 41598_2024_54140_MOESM1_ESM.docx]

**Supplemental Figure 1:** **Original Blot of Figure 1 C**

20 μg of whole protein lysates from CLB-Ga and CLB-Sedp cells treated or not for 24 h with 10 nM or 10 μM bortezomib associated or not with 10 μM tubacin were separated by SDS-PAGE electrophoresis and transferred onto nitrocellulose membranes. NT: Not treated; MT: Mock treated. Detection of K48 ubiquitinated proteins by anti-K48-linkage Specific Polyubiquitin mAb. HSC70 was used as a loading control. (A) Signals of K48 ubiquitinated proteins. (B) Signal of HSC70.

**Supplemental Figure 2:** **Original Blot of Figure 2 A and B**

15 μg of whole protein lysates from CLB-Ga and CLB-Sedp cells treated or not for 24 h with 10 nM or 10 μM bortezomib, and also 10 pM for CLB-Ga cells, were separated by SDS-PAGE electrophoresis and transferred onto nitrocellulose membranes. NT: Not treated; MT: Mock treated. Detection of Casp3 and PARP-1 by anti-Casp3 mAb and anti-Parp1 mAb, respectively. Ku80 was used as a loading control. (A-B) Signals of Casp3 and its cleaved form after exposition of the membrane during 10 s (A) or 1min (B). (C) Signals of Parp1 and its cleaved form after exposition of the membrane during 5 min. (D) Signal of Ku80 after exposition of the membrane during 30 s.
